# Common inherited variants of *PDCD1*, *CD274* and *HAVCR2* genes differentially modulate the risk and prognosis of adenocarcinoma and squamous cell carcinoma

**DOI:** 10.1007/s00432-023-04602-8

**Published:** 2023-02-09

**Authors:** Nafeesa Moksud, Marta Wagner, Konrad Pawełczyk, Irena Porębska, Beata Muszczyńska-Bernhard, Aneta Kowal, Andrzej Wiśniewski, Monika Kosacka, Julia Kończak, Paweł Karpiński, Dominik Frydryk, Anna Andrzejczak, Lidia Karabon, Piotr Kuśnierczyk, Monika Jasek

**Affiliations:** 1 Laboratory of Genetics and Epigenetics of Human Diseases, Hirszfeld Institute of Immunology and Experimental Therapy, Polish Academy of Sciences, Rudolfa Weigla 12, 53-114 Wrocław, Poland; 2 Lower Silesian Centre of Oncology, Pulmonology and Heamatology, Wrocław, Poland; 3 Department of Pulmonology and Lung Oncology, Wroclaw Medical University, Wrocław, Poland; 4 Laboratory of Immunogenetics and Tissue Immunology, Hirszfeld Institute of Immunology and Experimental Therapy, Polish Academy of Sciences, Wrocław, Poland; 5 Department of Genetics and Cell Physiology, University of Wroclaw, Wrocław, Poland; 6 Department of Genetics, Wroclaw Medical University, Wrocław, Poland

**Keywords:** SNPs, *PDCD1*, *CD274*, *HAVCR2*, NSCLC

## Abstract

**Background:**

To investigate the association between single nucleotide polymorphisms (SNPs) of *PDCD1*, *CD274*, and *HAVCR2* genes with the risk and outcomes of non-small cell lung cancer (NSCLC) subtypes: squamous cell lung cancer (LUSC) and lung adenocarcinoma (LUAD).

**Methods:**

TaqMan SNP genotyping assays or polymerase chain reaction-restriction fragment length polymorphism methods were used to determine genotypes of: *PDCD1:* rs36084323, rs7421861, rs11568821, rs2227981, rs10204525; *CD274*: rs822335, rs10815225, rs17718883, rs2297136, rs4742098, rs4143815; *HAVCR2*: rs10057302, rs1036199. Among 383 NSCLC patients, 112 were diagnosed with LUAD and 116 with LUSC. The control group consisted of 433 unrelated, cancer-free subjects.

**Results:**

A CC genotype of rs4143815 and GG genotype of rs4742098 were associated with two times higher risk of developing LUSC (CC vs. GG + GC, OR = 2.31; 95% CI = 1.32, 4.06; *P* = 0.003; GG vs. AA + AG, OR = 2.26; 95% CI = 1.17, 4.36; *P* = 0.016, respectively). Moreover, rs4143815 was an independent predictor of the age at diagnosis of LUAD. The carriers of C allele were diagnosed 4.81 years later (95% CI = 1.47, 8.15; *P* = 0.006) than patients with the GG genotype. The rs10057302 CA genotype was an independent predictor of overall survival in LUSC (adjusted HR = 0.13; 95% CI = 0.02, 0.93; *P* = 0.043). NSCLC carriers of rs11568821 T allele had almost double the risk of death (adjusted HR = 2.05; 95% CI = 1.28, 3.29; *P* = 0.003) compared to carriers of CC genotype.

**Conclusions:**

Our results provided additional evidence that SNPs of genes for PD-1, PD-L1 and TIM-3 differentially modulate the risk and prognosis of LUSC and LUAD.

**Supplementary Information:**

The online version contains supplementary material available at 10.1007/s00432-023-04602-8.

## Introduction

Non-small cell lung cancer (NSCLC) constitutes a heterogeneous group of cancers with three major histological subtypes: lung adenocarcinoma (LUAD) (40%), lung squamous cell carcinoma (LUSC) (25–30%) and large cell carcinoma (10%). LUAD and LUSC display many distinctive features related to histopathology, genomic and genetic architecture (Relli et al. 2019) and disease course, including tumour differentiation, stage-specific survival, underlying drivers and response to various therapeutic options (Faruki et al. 2017). Relli and colleagues have summarised the main differences between LUAD and LUSC, highlighting that the impact of prognostic determinants was frequently partially masked or even hidden when LUSC and LUAD were analysed together as NSCLC (Relli et al. 2019).

Immune checkpoint receptors such as PD-1 (programmed cell death protein 1, CD279; gene *PDCD1*) along with its ligands play a vital role in regulating the immune response. Under physiological conditions, immune checkpoints are a critical mechanism responsible for maintaining immunological homeostasis and preventing the development of autoimmune diseases (Lucibello et al. 2021; Andrews et al. 2019). In cancer, tumour cells employ immune checkpoint pathways to evade anti-tumour immune responses by the host’s immune system (Andrews et al. 2019).

The introduction of immunotherapy into clinical practice represents a significant development in cancer treatment, with the PD-1/PD-L1 (programmed death ligand 1; CD274; gene *CD274*) pathway being a primary target of this strategy in several cancers, including NSCLC. It has been shown that treatment with a single monoclonal antibody (mAb) directed against PD-1 or PD-L1 allowed a durable clinical response in about 18% of patients with advanced NSCLC (Datar et al. 2019). Despite the significant contribution of immune checkpoint inhibitors (ICIs) towards improving the outcomes of some patients, many others are resistant or develop adaptive resistance to immunotherapy or suffer disease progression despite treatment (Andrews et al. 2019; Curigliano et al. 2021). As a result, there is an urgent need for novel therapies or combinational strategies employing the blockade of other immune checkpoint receptors (IRs), such as TIM-3 (T-cell immunoglobulin domain and mucin domain-3, CD366, gene *HAVCR2*).

TIM-3 is frequently co-expressed with PD-1 (Curigliano et al. 2021), and its upregulation in NSCLC is considered a mechanism of adaptive resistance to PD-1 blockade (Andrews et al. 2019). Preclinical studies on animal models demonstrated that therapy involving simultaneous blockade of TIM-3 and PD-1 pathways improved anti-tumour response and effectively suppressed tumour growth, compared to when only one pathway was targeted (Andrews et al. 2019; Curigliano et al. 2021). These observations led to many clinical trials investigating the effectiveness of combining PD-1 and TIM-3 blockade in solid tumours (Acharya et al. 2020).

Genetic variants of genes for PD-1, PD-L1 and TIM-3 molecules have been investigated in several cancers to determine their potential association with disease risk and outcomes. Their association with response to immunotherapy and other types of treatment has also been studied, particularly for polymorphisms located in *PDCD1* and *CD274* genes. However, most of these studies were conducted mainly in East Asian cohorts. Since genotype frequencies and allele distribution of these variants differ significantly in various populations, more studies are needed to establish population-specific and “general” variants associated with risk, outcomes and response to treatment (Wagner et al. 2021). Moreover, only a few analyses considered NSCLC by histology.

Therefore, this study aimed to investigate the association between widely studied variants of *PDCD1*, *CD274* and *HAVCR2* genes with NSCLC risk and outcomes as well as by LUSC and LUAD subtypes separately. We also examined the contribution of haplotypes to the development and the course of NSCLC and its subtypes.

## Materials and methods

### NSCLC patients and control group

383 patients with a confirmed diagnosis of NSCLC were enrolled by the Department of Pulmonology and Lung Cancer, Wrocław Medical University and the Thoracic Surgery Centre, Lower Silesian Centre of Lung Disease, Wrocław, from 2005 to 2017. Table 1 presents baseline characteristics of NSCLC patients and the control group. Histological subtypes of 128 patients were unable to be determined as the diagnostic criteria of small biopsies and non-resection specimens were not established until 2011. The control group consisted of 433 unrelated cancer-free volunteers and blood donors. Written informed consent was obtained from all participants. The study was conducted following the Declaration of Helsinki and was approved by the Bioethics Committee of the Wroclaw Medical University (KB-586/2020 September 28, 2020; Addendum; October 13, 2020).

**Table 1:** Baseline characteristics of NSCLC patients and control group

Variables	Patients		Control group	
	Number	%	Number	%
Overall	383	100	433	100
Sex				
Male	276	72.06	313	72.29
Female	107	27.94	119	27.48
Missing value	0	0	1	0.23
Median age (range)^a^				
Male	64 (45–87)		32 (18–75)	
Female	62 (36–87)		33 (19–63)	
All	64 (36–87)		32 (19–75)	
Smoking status				
Ever smoker	318	83.03	98	22.63
Non-smoker	51	13.32	55	12.70
Missing value	14	3.65	280	64.67
Histological type				
LUAD	112	29.24		
LUSC	116	30.29		
LCC	21	5.49		
NEC	3	0.78		
ASC	3	0.78		
NOS	128	33.42		
Tumour stage				
I	86	22.45		
II	49	12.80		
III	118	30.81		
IV	123	32.11		
Missing value	7	1.83		
Therapy				
Surgery				
Yes	181	47.26		
No	200	52.22		
Missing value	2	0.52		
Chemotherapy				
Yes	250	65.27		
No	121	31.60		
Missing value	12	3.13		
Survival status				
Alive^b^	57	14.88		
Deceased	266	69.45		
Missing value	60	15.67		

*LUAD* adenocarcinoma; *LUSC* squamous cell carcinoma; *LCC* Large cell carcinoma; *ASC* adenosquamous cell carcinoma; *NEC* neuroendocrine carcinoma; *NOS* not otherwise specified

^a^Patients’ age at diagnosis

^b^Status on 31.12.2021

### Selection of single nucleotide polymorphisms (SNPs) and genotyping

We selected widely investigated genetic variants of *PDCD1* (rs36084323 C > T, rs7421861 A > G, rs11568821 C > T, rs2227981 A > G, rs10204525 C > T), *CD274* (rs822335 T > C, rs10815225 G > C, rs17718883 C > G, rs2297136 G > A, rs4742098 A > G, rs4143815 G > C), and *HAVCR2* ((rs10057302 C > A as a tagging SNP for rs10053538 C > A, LD *r*^2^ = 1.0) and rs1036199 C > A) in different types of cancer. A detailed description of these variants can be found in our review article, where we summarised the association between inherited variants in these genes and their functional relevance (Wagner et al. 2021). Allelic discrimination using TaqMan SNP Genotyping Assays Applied Biosystems™ and TaqMan™ Genotyping Master Mix Applied Biosystems™ (Cat. No.: 4351376, Cat. No.: 4381656, respectively, Thermo Fisher Scientific, Waltham, MA, USA) were used for genotyping all the examined SNPs according to the manufacturer’s protocol, except for rs2297136 and rs17718883, which were genotyped using polymerase chain reaction–restriction fragment length polymorphism (PCR–RFLP). The PCR products were digested with *Bsu*RI (*Hae*III) and *Hinf*I restriction enzymes, respectively (Cat. No: ER0151 and ER0801, respectively, Thermo Fisher Scientific, Waltham, MA, USA). Supplementary Table S1 contains a detailed list of genotyping assays used in this study. Primer sequences, annealing temperatures, restriction enzymes and restriction fragments length are listed in Supplementary Table S2. TaqMan SNP Genotyping Assays were run on ViiA 7 Real-Time PCR system and analysed with Quant Studio Real-Time PCR Software 2016 v.1.3 (Applied Biosystems, Thermo Fisher Scientific, Waltham, MA, USA). PCRs were carried out on T100™ Thermal Cycler (Bio-Rad, Hercules, CA, USA). Genotyping accuracy for all SNPs were verified by direct sequencing of a few samples representing homozygotes of two types and heterozygotes for each investigated SNP. These samples were used as reference samples in the subsequent genotyping experiments.

### In silico analysis

We applied several publicly available tools to indicate possible links between investigated germline variants in studied genes and the impact on development and course of the disease. dbSNP was the reference source for providing the SNP ID number, alleles and frequencies. RegulomeDB 2.0.3 (Boyle et al. 2012) and 3DSNP 2.0 tools (Quan et al. 2022) were used to examine the putative regulatory potential of SNPs. HaploRegv4.1 was applied for the functional annotation of SNPs as well as for analysis of Linkage Disequilibrium (LD) (Ward and Kellis 2012). Expression quantitative trait loci (eQTL) analysis was conducted using the Genotype-Tissue Expression (GTEx) portal V8 release. The University of Alabama at Birmingham Cancer (UALCAN) data analysis portal was used for the examination of hsa-mir-570 expression levels in LUAD and LUSC (Chandrashekar et al. 2017, 2022).

### Statistical analysis

The difference between genotype distribution in patients and the control group was analysed with a chi-squared test. The odds ratio and 95% confidence interval (95% CI) were applied as a measure of effect size. All analyses used the control group’s most frequent homozygote and allele as the reference category. Association between examined genetic variants and age at diagnosis was tested by linear regression model, adjusted for smoking and gender. Chi-squared or Fisher’s exact test was used to test the association between SNPs and clinical stage, lymph and distant metastases considering patients’ smoking status. Multivariable-adjusted hazard ratios (HRs) and 95% CI for all-cause survival were estimated using Cox proportional hazards models with adjustment for smoking and gender, using age at diagnosis as the time metric. Follow-up started at the day of NSCLC diagnosis and ended at the day of death or the end of follow-up (31/12/2021). Kaplan–Meier (KM) survival curves were estimated for each SNP between subgroups of surgical intervention (patients who underwent surgery vs. patients who did not undergo surgery). Haplotypes and their frequency were estimated using the likelihood-based expectation–maximization algorithm EM algorithm for *PDCD1* and *CD274* genes; *P* < 0.05 was considered statistically significant. Statistical analysis was conducted using R, version 4.1.3 (R Project for Statistical Computing) and STATA 14 (College Station, TX: StataCorp LLC).

## Results

### *PDCD1, CD274*, and *HAVCR2* genetic variants and susceptibility to NSCLC

Genotypes of selected SNPs were determined in 383 NSCLC and 433 unrelated cancer-free subjects. The detected allele frequencies for all SNPs were similar to the European population from the 1000 Genomes Project, as listed in dbSNP (Supplementary Table S3). There were no differences in genotype distribution between NSCLC patients and control subjects for any of our examined SNPs (Supplementary Tables S4–S6). As LUSC and LUAD are suspected of having different genetic backgrounds, we investigated the association between our selected SNPs and the risk of developing either subtype (Table 2, Supplementary Tables S7–S9 and S10–S12, respectively). We noticed a higher frequency of rs4143815 CC homozygotes among LUSC patients (19.0% vs. 9.2%). Rs4143815 CC genotype was associated with two times higher LUSC risk (CC vs. GG + GC, OR = 2.31; 95% CI = 1.32, 4.06; *P* = 0.003). Also, rs4742098 GG homozygotes were more frequent in LUSC patients (12.9% vs. 6.2%) and had two times higher LUSC development risk (GG vs. AA + GG, OR = 2.26; 95% CI = 1.17, 4.36; *P* = 0.016) (Table 2). In addition, we noticed an interesting, however, not statistically significant, association for rs11568821 C > T of *PDCD1*. Subjects carrying TT homozygotes might had more than three times higher risk of developing this NSCLC subtype compared to subjects with CC or CT genotype (TT vs. CC + CT, OR = 3.12; 95% CI = 0.88, 11.02; *P* = 0.084). We did not notice any significant results for any examined SNPs for LUAD.

**Table 2:** Association of *PDCD1* and *CD274* variants and the risk of LUSC

Gene variant		LUSC patients *n* = 116		Controls *n* = 433		OR	95% CI	Patients vs. controls *P* value
*PDCD1* (-strand)		*n*	%	*n*	%			
rs11568821 C > T c.627 + 189 intron 4 PD-1.3	CC	95	81.9	362	83.6	1	Ref	*Χ*^2^_df=2_ = 2.9886 *P* = 0.2244
	CT	17	14.7	66	15.2	1.00	0.56–1.77	
	TT	4	3.4	5	1.2	3.11	0.88–11.02	
HWE		*P*_HWE_ = 0.0104		*P*_HWE_ = 0.3175				
	CC + CT	112	96.6	428	98.8	1	Ref	*Χ*^2^_df=1_ = 2.9792 *P* = 0.0843
	TT	4	3.4	5	1.2	3.12	0.88–11.02	
*CD274*								
rs4143815 G > C c.*395 exon 7 (3′UTR)	GG	50	43.1	206	47.6	1	Ref	*Χ*^2^_df=2_ = 8.6590 *P* = 0.0132
	GC	44	37.9	187	43.2	0.97	0.62–1.52	
	CC	22	19.0	40	9.2	2.27	1.25–4.14	
HWE		*P*_HWE_ = 0.0362		*P*_HWE_ = 0.7938				
	GG + GC	94	81.0	393	90.8	1	Ref	*Χ*^2^_df=1_ = 8.6262 *P* = 0.0033
	CC	22	19.0	40	9.2	2.31	1.32–4.06	
rs4742098 A > G c.*2635A exon 7 (3′UTR)	AA	61	52.6	230	53.1	1	Ref	*Χ*^2^_df=2_ = 6.2495 *P* = 0.0439
	AG	40	34.5	176	40.7	0.86	0.55–1.34	
	GG	15	12.9	27	6.2	2.11	1.07–4.18	
HWE		*P*_HWE_ = 0.0504		*P*_HWE_ = 0.3827				
	AA + AG	101	87.1	406	93.8	1	Ref	*Χ*^2^_df=1_ = 5.7947 *P* = 0.0161
	GG	15	12.9	27	6.2	2.26	1.17–4.36	

Statistically significant (*P* < 0.05)

SNP alleles are reported in the Forward orientation in agreement with the dbSNP database. The most frequent homozygote in the control group was used as the reference genotype

*OR* odds ratio; *CI* confidence interval; *ref.* reference genotype; *HWE* test for Hardy–Weinberg equilibrium

### *PDCD1, CD274*, and *HAVCR2* genetic variants and NSCLC prognosis

Next, we investigated the prognostic outcomes of selected SNPs. First, we checked if any of selected SNPs were associated with age at diagnosis in NSCLC as a whole group and by LUSC and LUAD separately. Rs4143815 of *CD274* appeared to be an independent predictor of the age at diagnosis of LUAD. LUAD patients carrying C allele (GC + CC) were diagnosed 4.81 years later than patients with GG genotype (parameter = 4.81; 95% CI = 1.47, 8.15; *P* = 0.006) (Table 3).

**Table 3:** Prognostic significance of *CD274*, *PDCD1*, and *HAVCR2* variants

SNV gene	LUAD	*n*		Parameter [year]^a^		95% CI		*P*^a^
rs4143815 G > C *CD274*	GG	47		Ref.				
	GC	49		5.54		2.04	9.04	0.002
	CC	12		1.80		−3.77	7.37	0.528
	GG	47		Ref.				
	GC + CC	61		4.81		1.47	8.15	0.006
rs10057302 C > A *HAVCR2*	**LUSC**	*n*	Deaths	HR		95% CI		*P*^b^
	CC	83	64	1		Ref.		
	CA	5	1	0.13		0.02	0.93	0.043
rs11568821 C > T *PDCD1*	**NSCLC Operated patients**	*n*	Deaths	HR		95% CI		*P*^b^
	CC	134	85	1		Ref.		
	CT	27	22	1.97		1.21	3.22	0.006
	TT	2	2	3.43		0.82	14.37	0.091
	CC CT + TT	134 29	85 24	1 2.05		Ref. 1.28	3.29	0.003
rs10057302 C > A *HAVCR2*	**NSCLC**		*n*	M0	M1	Mx	*P*^c^	
	CC_NON-SMOKER_		44	22 (50.00%)	16 (36.36%)	6 (13.64%)	0.009	
	CC_SMOKER_		292	187 (64.04%)	95 (32.53%)	10 (3.42%)		
	CA_SMOKER_		24	17 (70.83%)	4 (16.67%)	3 (12.50%)		
rs1036199 C > A *HAVCR2*	**NSCLC**		*n*	M0	M1	Mx	*P*^c^	
	AC_NON-SMOKER_		17	6 (35.29%)	10 (58.82%)	1 (5.88%)	0.002	
	AA_NON-SMOKER_		31	19 (61.29%)	6 (19.35%)	6 (19.35%)		
	CC_SMOKER_		15	7 (46.67%)	8 (53.33%)	0 (0.00%)		
	AC_SMOKER_		93	64 (68.82%)	27 (29.03%)	2 (2.15%)		
	AA_SMOKER_		207	132 (63.77%)	64 (30.92%)	11 (5.31%)		

*HR* hazard ratio; *CI* confidence intervals; *M0* cancer has not spread to other parts of the body; *M1* cancer has spread to other parts of the body: *Mx* metastasis cannot be measured

*P*^a^ linear regressions adjusted for smoking and gender; statistically significant (*P* < 0.05)

*P*^b^ Cox proportional hazard models adjusted for smoking and gender; statistically significant (*P* < 0.05)

*P*^c^ chi-squared or Fisher’s exact test; statistically significant (*P* < 0.05)

^a^Difference in the age at diagnosis

**Fig. 1:**
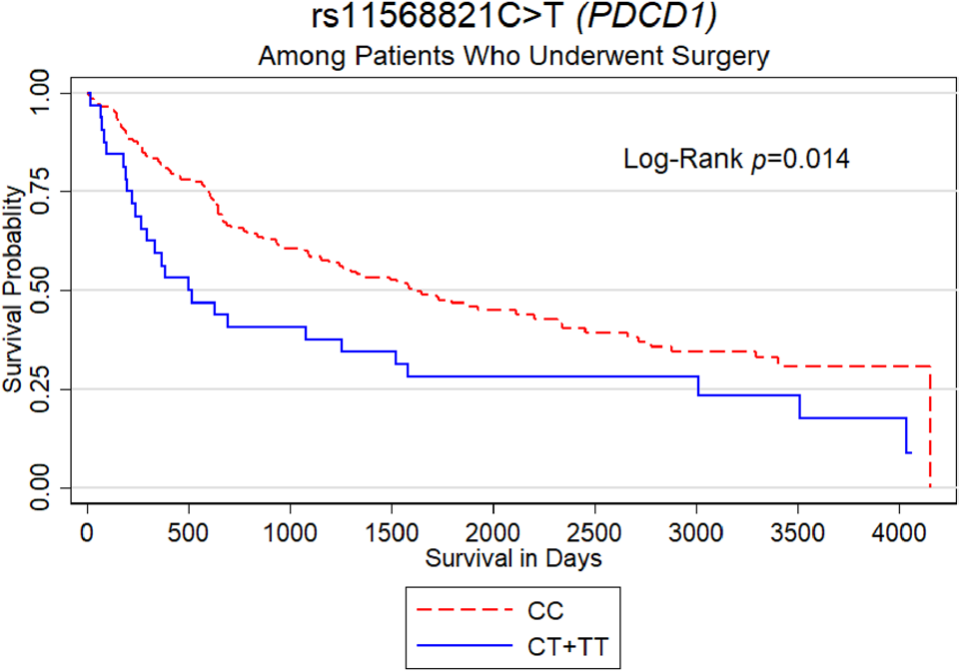
The rs11568821 germline variant of the *PDCD1* gene predicts the overall survival of NSCLC patients who have undergone surgery. The carriers of allele T (CT + TT) had almost double the risk of all-cause mortality (aHR = 2.052; 95% CI = 1.281, 3.287; *P* = 0.003). Cox proportional hazard models were adjusted for smoking and gender

We then examined the association between investigated SNPs and: (1) cancer clinical stage, (2) overall survival (OS), (3) OS and surgery status, (4) lymph nodes metastasis, and (5) distal metastasis. These analyses were performed for NSCLC, LUSC and LUAD, adjusting for smoking and gender where applicable. We found that CA genotype at *HAVCR2* rs10057302 might be an independent predictor of OS in LUSC. Patients with this genotype had about 87% lower chance of death at any time than patients with CC genotype (aHR = 0.13; 95% CI = 0.02, 0.93; *P* = 0.043) (Table 3). Among NSCLC patients who underwent surgery, rs11568821 T allele carriers (CT + TT) had almost double the risk of all-cause mortality (aHR = 2.05; 95% CI = 1.28, 3.29; *P* = 0.003) compared to patients carrying CC genotype (Fig. 1, Table 3).

Moreover, distal metastasis was present in a lower percentage of NSCLC patients who were smokers and *HAVCR2* rs10057302 carriers of CA genotype than patients with CC genotype irrespective of smoking status (CA smokers 16.67% vs. CC Non-smokers 36.36%, CC smokers 32.53%; *P* = 0.009) (Table 3). For *HAVCR2* rs1036199, we observed a higher percentage of patients with distal metastasis among non-smoking C allele carriers (AA non-smoker 19.35% vs. CA smokers 58%) and smokers with CC genotype (AA smokers 29.03%, AC smokers 29.03% vs. CC smokers 53.33%) (Table 3).

### Predictive values of *PDCD1* and *CD274* haplotypes in NSCLC

Supplementary Tables S13 and S14 show the distribution of the most frequent haplotypes in the Polish population for *PDCD1* and *CD274*. The specific combination of adjacent SNPs could be necessary for revealing a particular phenotype; therefore, haplotype analysis may allow the determination of such distinctive SNP composition (Ek 2007). Hence, we also examined the association between haplotypes and NSCLC risk and its subtypes, as well as clinicopathological data, such as age at diagnosis and OS. None of the estimated haplotypes was associated with NSCLC risk. However, the *CD274* haplotype rs822335*C-rs10815225*G-rs17718883*C-rs2297136*A-rs4143815*G-rs4742098*A (reference haplotype: rs822335*C-rs10815225*G-rs17718883*C-rs2297136*G-rs4143815*G-rs4742098*A) might decrease the age at diagnosis of NSCLC on average by 1 year and 5 months after adjustment for smoking (Parameter =  − 1.51; 95% CI =  − 2.73, − 0.28; *P* = 0.016) (Supplementary Table S15). The haplotype associated with earlier age at diagnosis differs from the reference haplotype only by A allele of rs2297136. This SNP is located within the binding site for hsa-mir-296-5p, with A allele affecting binding between hsa-mir-296-5p and *CD274* mRNA. Accordingly, in the reporter assay, G allele was associated with lower luciferase activity in relation to A allele (Du et al. 2017). In gastric cancer, AA and AG genotypes of rs2297136 were significantly more frequent among patients with positive PD-L1 expression assayed by immunohistochemistry (IHC) (Wu et al. 2019). Of note, we observed a better prognosis in terms of survival for operated NSCLC patients having G allele, but after adjustment for smoking and gender, this correlation lost statistical significance (Supplementary Figure 1). By contrast, the haplotype rs822335*T-rs10815225*G-rs17718883*C-rs2297136*A-rs4143815*G-rs4742098*A increased the age of diagnosis of NSCLC by approximately 2 years (Parameter = 1.98; 95% CI = 0.40, 3.56; *P* = 0.014) (Supplementary Table S15). This haplotype had T allele at rs822335 in addition to allele A at rs2297136. The impact of rs822335 on PD-L1 expression was investigated in tissue samples of NSCLC patients by Krawczyk et al. (2019) and Grenada et al. (2021). These authors detected a higher percentage of tumour cells presenting PD-L1 assessed by IHC in samples from patients with CC genotype compared to CT or TT genotypes (Krawczyk et al. 2019; Grenda et al. 2021). We carried out in silico analysis with RegulomeDB and 3DSNP 2.0 databases which showed that rs822335 was annotated with the enhancer state in the A549 cell line and Lung Fibroblast Primary Cells (NHLF), however, any potential binding site for transcription factor related to rs822335 was revealed.

Two haplotypes of *CD274* decreased the age at diagnosis of LUSC. The abovementioned haplotype rs822335*C-rs10815225*G-rs17718883*C-rs2297136*A-rs4143815*G-rs4742098*A lowered the age at diagnosis by about 3 years (Parameter =  − 3.26; 95% CI =  − 5.45, − 1.07; *P* = 0.004) and haplotype rs822335*T-rs10815225*G-rs17718883*C-rs2297136*A-rs4143815*C-rs4742098*G by nearly 2 years (Parameter =  − 1.83; 95% CI =  − 3.44, − 3.22; *P* = 0.026) (Supplementary Table S16). It is worth noting that the last haplotype had the opposite effect in LUAD, where it was associated with higher age at diagnosis by about 6 years (Parameter = 6.13; 95% CI = 3.42, 8.84; *P* < 0.001) (Supplementary Table S17).

When analysing the association between haplotypes and OS, we noticed that rs36084323*C-rs7421861*G-rs11568821*T-rs2227981*G-rs10204525*C haplotype of *PDCD1* after adjustment for smoking increased the probability of death among NSCLC patients who underwent surgery to about 70% (HR = 1.70; 95% CI = 1.13, 2.57; *P* = 0.011) in relation to the reference haplotype: rs36084323*C-rs7421861*A-rs11568821*C-rs2227981*A-rs10204525*C (Supplementary Table S18). The haplotype associated with a worse prognosis contained T allele of rs11568821, which in our study was associated with worse survival of patients undergoing surgery, and the G allele of rs7421861, associated with a higher overall cancer risk (Hashemi et al. 2019; Zhang et al. 2021). These haplotypes also differ regarding rs2227981 (also known as PD-1.5 or + 7785). The association between A allele and a higher percentage of PD-1^+^CD4^+^ T cells was detected in Japanese patients with autoimmune type I diabetes (Fujisawa et al. 2015). The data relating to association of rs2227981 with NSCLC are conflicting: Yin and colleagues (2014) observed that A allele was associated with lower NSCLC risk, but no such association was detected by Ma and colleagues (2015). Similarly, an Iranian study did not find associations between rs2227981 and NSCLC (Piredelkhosh et al. 2018). Further studies are needed to confirm the observed correlations between particular haplotypes and clinical outcomes. It is also essential to carry out functional studies to reveal the biological implication of analysed genetic variants as a single locus and variants interacting in a particular combination.

## Discussion

Our study aimed to investigate whether SNPs present in genes for PD-1, PD-L1 and TIM-3 molecules could predispose to NSCLC development, progression, and affect patient outcomes. Being aware of possible etiological differences between the LUAD and LUSC, we also examined if selected variants were associated with either subtype.

As most published reports investigated the association between genetic variants of immune checkpoints without distinguishing between LUAD and LUSC, we have also used this approach; however, we did not observe any significant association for NSCLC as a whole group. In our study, CC genotype of rs4143815 appeared to be associated exclusively with a higher LUSC risk. This variant is in the 3’UTR of *CD274* within the seed region of the hsa-mir-570 binding site, with rs4143815 allele C predicted to affect the interaction between hsa-mir-570 and *CD274* mRNA (Wang et al. 2013). Functional relevance of this allele was confirmed in luciferase assays, where C allele was associated with higher luciferase activity in gastric cancer (Wang et al. 2013) and NSCLC (Lee et al. 2017). Moreover, Yeo and colleagues found an association between elevated expression of PD-L1 protein evaluated by IHC and CC genotype of rs4143815 in NSCLC samples (Yeo et al. 2017). It is worth mentioning that increased levels of hsa-mir-570 were detected in NSCLC tumour tissue in relation to adjacent non-malignant tissue (Tong et al. 2015). Since we observed the different impact of rs4143815 on LUSC and LUAD, we checked if hsa-mir-570 is differently expressed in the microenvironment of these two subtypes of NSCLC. As presented in Supplementary Fig. S2, hsa-mir-570 expression was significantly higher in LUSC (Chandrashekar et al. 2017, 2022). Based on available data, it can be assumed with a high probability that NSCLC patients with rs4143815 CC genotype will have elevated PD-L1 expression in tumour tissue, indicating better responses to mAbs blocking the interaction between PD-1 and PD-L1 (Nomizo et al. 2017). This relation has been already described in literature: Nomizo and colleagues investigated the relationship between rs4143815 genotypes and response to nivolumab (anti-PD-1 mAbs) and found rs4143815 allele C carriers (GC and CC genotypes) treated with nivolumab showed significantly better clinical response than patients with GG genotype, with the highest objective response rate observed for rs4143815 CC genotype as well as the longest progression-free survival (PFS) (Nomizo et al. 2017). In line with this observation, a significantly shorter PFS associated with rs4143815 GG in NSCLC patients treated with nivolumab was observed in a study by Del Re and colleagues (Re et al. 2021). So far, the largest study investigating the response to ICIs in relation to rs4143815 was performed by Minari and colleagues on Italian patients with metastatic NSCLC (Minari et al. 2022). This group did not find a significant correlation between rs4143815 genotypes and clinical outcomes such as PFS or OS. Nevertheless, they reported a significant association between allele C of rs4143815 (GC and CC genotypes) and long-lasting responses to ICIs treatment (Minari et al. 2022).

To the best of our knowledge, no reports have shown an association between rs4143815 variant and NSCLC risk. In work by Du and colleagues, genotypes of this SNP were not associated with risk or analysed clinical covariates (Du et al. 2017). In terms of clinical outcomes, Lee and colleagues reported the association between rs4143815 genotype GG and significantly worse OS in NSCLC under the recessive model. Analysis by histology revealed that this association appeared to be significant only in LUAD (Lee et al. 2017). Yeo et al. obtained a similar result for rs4143815 GG genotype. These authors observed that this genotype was related to a shorter disease-free survival and OS among LUAD patients; however, this association was not significant (Yeo et al. 2017). In our study, rs4143815 genotype GG was not associated with OS either for NSCLC or after stratification by histology; however, this genotype correlated significantly with the earlier onset of LUAD in relation to C allele carriers at this site.

In our study, rs4742098, another 3′UTR variant of *CD274*, was also associated with LUSC, with GG genotype significantly increasing the risk of developing this subtype. This variant is located within the seed region of hsa-mir-138, and its functional relevance was confirmed by luciferase assay where allele G, predicted to impact the binding of hsa-mir-138 to 3’UTR of *CD274* mRNA, was associated with higher luciferase activity in contrast to decreased activity demonstrated for allele A (Du et al. 2017). Hence, it may be assumed that the substitution of A to G in rs4742098 will be responsible for the higher expression of PD-L1. Accordingly, Du and colleagues observed that rs4742098 AG genotype was associated with a higher NSCLC risk in relation to AA genotype; surprisingly GG genotype did not show this effect (Du et al. 2017).

It is established that surgery is the best cure for early-stage NSCLC; however, a significant proportion of operated patients die from cancer relapse. Considerable variability in survival exists even among NSCLC patients within the same stage, indicating that host genetic factors may determine prognosis (Shin et al. 2018).

Among NSCLC patients who underwent surgery in our cohort, rs11568821 T allele carriers had a significantly higher probability of death compared to patients with CC genotype. Two more studies investigated rs11568821 in NSCLC, one in a Chinese (Ma et al. 2015), another in an Iranian population (Piredelkhosh et al. 2018). The association between rs11568821 genotypes and the risk or outcomes of NSCLC was not found in either study, similar to our results. These reports did not stratify analyses by histology. In a retrospective study, melanoma patients carrying CC homozygotes in rs11568821 had better responses to nivolumab or pembrolizumab than patients carrying T allele (CT and TT genotypes), showing C allele significantly associated with longer median PFS (Parakh et al. 2021). It is worth mentioning that rs11568821, according to RegulomeDB, has been annotated with chromatin states related to the strong transcription in different subpopulations of T cells and lung tissue. It can also be considered a *cis*-eQTL variant for the *PDCD1* gene in lung tissue according to GTEx, with T allele associated with higher expression of *PDCD1* (Supplementary Figure 3). Therefore, it can be speculated that the higher mortality rate among operated rs11568821 T allele carriers in our study may be due to insufficient response from the immune system against the remaining tumour cells resulting from higher PD-1 expression, and thus a weaker response from T lymphocytes. Due to the low frequency of T allele in our and other populations, a lack of sufficient data about the impact of rs11568821 on PD-1 expression/activity and only two case–control studies related to this variant in NSCLC, our results must be considered with due caution. Further investigation needs to be conducted to evaluate the biological relevance of *PDCD1* rs11568821, and its association with NSCLC histology, risk and/or outcomes must be validated in larger groups of control subjects and patients.

We investigated two variants of the *HAVCR2* gene, rs10057302 and rs1036199. We found that patients carrying CA genotype of rs10057302 (our tag SNP for rs10053538 C > A) had better OS than patients with CC genotype. We observed this correlation only for patients with LUSC. In addition, we noticed that NSCLC patients who smoked and were rs10057302 CA genotype carriers were less likely to develop distal metastasis compared to those with CC genotype, which may explain the better prognosis observed in our patients with LUSC. To our knowledge, apart from our study, only Bai and colleagues investigated rs10053538 in NSCLC (Bai et al. 2013) but did not find any associations. However, the authors did not stratify patients by histology.

Bai et al. also examined rs1036199 and found that CA genotype was associated with a higher NSCLC risk and shorter survival compared to AA genotype (Bai et al. 2013). We did not find an association between rs1036199 genotypes and NSCLC or its subtypes. Nonetheless, we noticed a higher percentage of patients with distal metastasis among non-smokers with CA genotype and smokers with CC genotype. The association of CA genotype with distal metastasis was also observed in breast cancer and renal cell carcinoma (Cheng et al. 2017; Cai et al. 2012).

To the best of our knowledge, our study is the first one comprehensively analysing the impact of frequently investigated common inherited variants of genes for PD-1, PD-L1 and TIM-3 in European NSCLC patients.

The main limitation of our study is a relatively low number of patients in the LUSC and LUAD groups, which might impact the significance level obtained for examined SNPs, especially those with low minor allele frequencies (MAFs). Moreover, since our control group largely consisted of blood donors, there was a significant difference between the median age of patients and control subjects. Further research is therefore required. Nonetheless, our study provides strong evidence that germline variants in genes for immune checkpoint molecules may impact NSCLC risk and outcomes and identifying these variants may be helpful in clinical practice.

In summary, our results are in line with the postulate that LUAD and LUSC should be analysed as two distinct biological subtypes (Relli et al. 2019; Faruki et al. 2017). This distinction may be essential when planning the management of patients with NSCLC. Taking this difference into consideration, further research may fine-tune findings which can improve risk and prognosis prediction and even guide new treatment modalities.

## Supplementary Information

Below is the link to the electronic supplementary material.

Supplementary file1 (DOCX 1808 KB)

## Data Availability

The datasets generated and analysed during the current study are available from the corresponding author on reasonable request.

## Abbreviations

NSCLC: Non-small cell lung cancer
LUAD: Lung adenocarcinoma
LUSC: Lung squamous cell carcinoma
PD-1: Programmed cell death protein 1
PD-L1: Programmed death ligand 1
mAb: Monoclonal antibody
ICIs: Immune checkpoint inhibitors
IRs: Immune checkpoint receptors
TIM-3: T-cell immunoglobulin domain and mucin domain-3
SNPs: Single nucleotide polymorphisms
PCR–RFLP: Polymerase chain reaction–restriction fragment length polymorphism
LD: Linkage disequilibrium
eQTL: Expression quantitative trait loci
GTEx: The Genotype-tissue expression
UALCAN: The University of Alabama at Birmingham Cancer Data Analysis Portal
95% CI: 95% Confidence interval
HR: Hazard ratios
KM curve: Kaplan–Meier curve
OS: Overall survival
aHR: Adjusted hazard ratio
mRNA: Messenger RNA
IHC: Immunohistochemistry
NHLF: Normal human lung fibroblast
PFS: Progression-free survival
MAF: Minor allele frequency
