# Supplementary material for: Common inherited variants of PDCD1, CD274 and HAVCR2 genes differentially modulate the risk and prognosis of adenocarcinoma and squamous cell carcinoma
Source: J Cancer Res Clin Oncol. 2023 Feb 9;149(9):6381–90. doi: 10.1007/s00432-023-04602-8 (PMC10356891; doi:10.1007/s00432-023-04602-8)
Supplement: Supplementary file 1 — Supplementary file1 (DOCX 1808 KB) [file 432_2023_4602_MOESM1_ESM.docx]

**Supplementary Table 1**. Assay ID of TaqMan probes used for genotyping of selected SNPs

| **SNP** | **LOCATION GRCh38** | **ASSAY ID** |
| --- | --- | --- |
| ***PDCD1*** |  |  |
| rs36084323 | 2:241859444 | C__57931321_10 |
| rs11568821 | 2:241851760 | C__57931290_10 |
| rs2227981 | 2:241851121 | C__57931286_20 |
| rs10204525 | 2:241850169 | C___172862_20 |
| rs7421861 | 2:241853198 | C__26891639_10 |
| ***CD274*** |  |  |
| rs822335 | 9:5448218 | C_7590674_10 |
| rs4143815 | 9:5468257 | C__31941235_10 |
| rs10815225 | 9:5450497 | C___1348557_30 |
| rs4742098 | 9:5470497 | C__27957750_10 |
| **TIM-3** |  |  |
| rs10057302 | 5:157087601 | C__29607693_10 |
| rs1036199 | 5:157104725 | C___2082038_1_ |

**Supplementary Table 2.** PCR-RFLP.

| **SNP** | **Forward Primer (5’-3’)**  **Reverse primer (5’-3’)** | **T_a_** | **Restriction fragment lengths** | **Restriction enzyme** |
| --- | --- | --- | --- | --- |
| rs2297136 | F-CCTGCTGCTTTCTCTCATTTC  R-GCTCCCTGTTTGACTCCATC | 60 | AA 284,72 bp  GA 284, 250,72,34 bp  GG 250,72,34 bp | HaeIII |
| rs17718883 | F-TTTGAATTGAATTGAGGCAGAG  R-ACCTGGGATGACCAATTCAG | 63 | CC 378 ,91 bp  CG 378, 222,156,91bp  GG 222,156,91 bp | HinfI |

T_a_-annealing temperature

**Supplementary Table S3.** Allele frequency of investigated SNPs in different populations.

|  | **Allele frequency** | | | | |
| --- | --- | --- | --- | --- | --- |
|  | **Present study**  **Poles** | **1000 Genomes dbSNP** | | | |
|  |  | **Europeans** | **East Asians** | **South Asians** | **Africans** |
| ***PDCD1*** |  |  |  |  |  |
| **rs36084323 C>T** | C=0.979 T=0.021 | C=0.9851 T=0.0149 | C=0.5317 T=0.4683 | C=0.943 T=0.057 | C=0.9584 T=0.0416 |
| **rs7421861 A>G** | A=0.655  G=0.345 | A=0.6481 G=0.3519 | A=0.8274 G=0.1726 | A=0.618 G=0.382 | A=0.7731 G=0.2269 |
| **rs11568821 C>T** | C=0.912  T=0.088 | C=0.8777 T=0.1223 | C=1.0000 T=0.0000 | C=0.970 T=0.030 | C=0.9909 T=0.0091 |
| **rs2227981 A>G** | A=0.585  G=0.415 | A=0.4016  G=0.5984 | A=0.2679  G=0.7321 | A=0.241 G=0.759 | A=0.4470  G=0.5499 |
| **rs10204525 C>T** | C=0.8834  T=0.1166 | C=0.8837 T=0.1163 | C=0.3403 T=0.6597 | C=0.807 T=0.193 | C=0.6089 T=0.3911 |
| ***CD274*** |  |  |  |  |  |
| **rs822335 T>C** | T=0.359  C=0.641 | T=0.3678 C=0.6322 | T=0.6587 C=0.3413 | T=0.349 C=0.651 | T=0.3903 C=0.6097 |
| **rs10815225 G>C** | G=0.885  C=0.115 | G=0.8718 C=0.1282 | G=0.9167 C=0.0833 | G=0.816 C=0.184 | G=0.7269 C=0.2731 |
| **rs17718883 C>G** | C=0.998  G=0.002 | C=0.9920 G=0.0080 | C=1.0000 G=0.0000 | C=1.000 G=0.000 | C=1.0000 G=0.0000 |
| **rs2297136 G>A** | G=0.529  A=0.471 | G=0.4533 A=0.5467 | G=0.1895 A=0.8105 | G=0.329 A=0.671 | G=0.3419 A=0.6581 |
| **rs4742098 A>G** | A=0.746  G=0.254 | A=0.7008 G=0.2992 | A=0.4643 G=0.5357 | A=0.808 G=0.192 | A=0.8865 G=0.1135 |
| **rs4143815 G>C** | G=0.692  C=0.308 | G=0.6700 C=0.3300 | G=0.4286 C=0.5714 | G=0.803 C=0.197 | G=0.9879 C=0.0121 |
| ***HAVCR2*** |  |  |  |  |  |
| **10057302 C>A** | C=0.962  A=0.038 | C=0.9533 A=0.0467 | C=0.9425 A=0.0575 | C=0.881 A=0.119 | C=0.9728 A=0.0272 |
| **rs1036199 C>A** | C=0.199 A=0.801 | C=0.1819 A=0.8181 | C=0.0159 A=0.9841 | C=0.034 A=0.966 | C=0.2110 A=0.7890 |

**Supplementary Table S4**. Genotype distribution of the *PDCD1* (2q37.3) single nucleotide polymorphisms (SNPs) in NSCLC patients and controls.

| ***PDCD1***  **(-strand)** | | **Patients**  **n = 383** | | **Controls**  **n= 433** | | **OR** | **95% CI** | **Patients vs. Controls** |
| --- | --- | --- | --- | --- | --- | --- | --- | --- |
|  |  | n | % | n | % |  |  |  |
| **rs36084323* C>T**  535 bp of *PDCD1*  **PD-1.1 (-606)** | CC | 372 | 97.1 | 415 | 95.8 | 1 | ref. | Χ^2^ _df=1_ = 0.9778  *P* = 0.3227 |
|  | CT | 11 | 2.9 | 18 | 4.2 | 0.69 | 0.33-1.47 |  |
| **HWE** |  | *P*_HWE_ = 0.7755 | | *P*_HWE_ = 0.6587 | |  |  |  |
| **rs7421861 A>G**  c.77-218 intron 1 | AA | 153 | 39.9 | 182 | 42.0 | 1 | ref. | Χ^2^ _df=2_ = 1.2518  *P* = 0.5348 |
|  | AG | 178 | 46.5 | 203 | 46.9 | 1.04 | 0.78-1.40 |  |
|  | GG | 52 | 13.6 | 48 | 11.1 | 1.29 | 0.82-2.01 |  |
| **HWE** |  | *P*_HWE_ = 0.9840 | | *P*_HWE_ = 0.4419 | |  |  |  |
| **rs11568821 C>T**  c.627+189 intron 4  **PD-1.3** | CC | 311 | 81.2 | 362 | 83.6 | 1 | ref. | Χ^2^ _df=2_ = 0.8953  *P* = 0.6391 |
|  | CT | 66 | 17.2 | 66 | 15.2 | 1.16 | 0.8-1.69 |  |
|  | TT | 6 | 1.6 | 5 | 1.2 | 1.38 | 0.44-4.32 |  |
| **HWE** |  | *P*_HWE_ = 0.2570 | | *P*_HWE_ = 0.3175 | |  |  |  |
| **rs2227981 A>G**  c.804 exon 5 (p.Ala268=)  **PD-1.5** | GG | 128 | 33.4 | 147 | 33.9 | 1 | ref. | Χ^2^ _df=2_ = 0.1310  *P* = 0.9366 |
|  | GA | 193 | 50.4 | 213 | 49.2 | 1.04 | 0.77-1.41 |  |
|  | AA | 62 | 16.2 | 73 | 16.9 | 0.98 | 0.65-1.47 |  |
| **HWE** |  | *P*_HWE_ = 0.4491 | | *P*_HWE_ = 0.7798 | |  |  |  |
| **rs10204525 C>T** c.*889 (3'UTR) **PD-1.6** | CC | 307 | 80.2 | 340 | 78.5 | 1 | ref. | Χ^2^ _df=2_ = 0.3581  *P* = 0.8361 |
|  | CT | 70 | 18.3 | 85 | 19.6 | 0.91 | 0.64-1.30 |  |
|  | TT | 6 | 1.6 | 8 | 1.8 | 0. 85 | 0.30-2.38 |  |
| **HWE** |  | *P*_HWE_ = 0.3892 | | *P*_HWE_ = 0.3249 | |  |  |  |

SNP alleles are reported in the **Forward** orientation in agreement with the dbSNP database; **reference genotype** - the most frequent homozygote in the control group; Abbreviations: OR, odds ratio; CI, confidence intervals, HWE, test for Hardy-Weinberg equilibrium; *LD z rs2227982;

**Supplementary Table S5**. Genotype distribution of the *CD274* (9p24.1) single nucleotide polymorphisms (SNPs) in NSCLC patients and controls.

| ***CD274***  **(+strand)** | | **Patients**  **n= 383** | | **Controls**  **n = 433** | | **OR** | **95% CI** | **Patients vs. Controls** |
| --- | --- | --- | --- | --- | --- | --- | --- | --- |
|  |  | n | % | n | % |  |  |  |
| **rs822335 T>C**  2.3 kb 5’ of  *CD274* | CC | 150 | 39.2 | 178 | 41.1 | 1 | ref. | Χ^2^ _df=2_ = 0.3376  *P* = 0.8447 |
|  | CT | 183 | 47.8 | 199 | 46.0 | 1.09 | 0.81-1.47 |  |
|  | TT | 50 | 13.1 | 56 | 12.9 | 1.06 | 0.68-1.64 |  |
| **HWE** |  | *P*_HWE_ = 0.6174 | | *P*_HWE_ = 0.9739 | |  |  |  |
| **rs10815225 G>C**  (-114) 5bp 5' of  *CD274* | GG | 301 | 78.6 | 345 | 79.7 | 1 | ref. | Χ^2^ _df=2_ = 3.9741  *P* = 0.1371 |
|  | GC | 78 | 20.4 | 76 | 16.6 | 1.18 | 0.83-1.67 |  |
|  | CC | 4 | 1.0 | 12 | 2.8 | 0.41 | 0.14-1.23 |  |
| **HWE** |  | *P*_HWE_ = 0.6713 | | *P*_HWE_ = 0.0034 | |  |  |  |
| **rs17718883 C>G**  c.437 exon 4 (p.Pro146Arg) | CC | 369 | 99.2 | 423 | 99.5 | 1 | ref. | Χ^2^ _df=1_ = 0.3585  *P* = 0.5493 |
|  | GC | 3 | 0.8 | 2 | 0.50 | 1.60 | 0.31-8.18 |  |
| **HWE** |  | *P*_HWE_ = 0.9378 | | *P*_HWE_ = 0.9612 | |  |  |  |
| **rs2297136G>A**  c.*93G>A exon 7 (3'UTR) | GG | 96 | 25.3 | 112 | 25.9 | 1 | ref. | Χ^2^ _df=2_ = 0.0932  *P* = 0.9545 |
|  | GA | 209 | 55.0 | 233 | 53.9 | 1.05 | 0.75-1.45 |  |
|  | AA | 75 | 19.7 | 87 | 20.1 | 1.01 | 0.67-1.52 |  |
| **HWE** |  | *P*_HWE_ = 0.0439 | | *P*_HWE_ = 0.0871 | |  |  |  |
| **rs4143815 G>C**  c.*395 exon 7 (3'UTR) | GG | 170 | 44.4 | 206 | 47.6 | 1 | ref. | Χ^2^ _df=2_ = 2.2039  *P* = 0.3322 |
|  | GC | 166 | 43.3 | 187 | 43.2 | 1.08 | 0.80-1.44 |  |
|  | CC | 47 | 12.3 | 40 | 9.2 | 1.42 | 0.89-2.26 |  |
| **HWE** |  | *P*_HWE_ = 0.5124 | | *P*_HWE_ = 0.7938 | |  |  |  |
| **rs4742098 A>G** c.*2635A exon 7 (3'UTR) | AA | 202 | 52.7 | 230 | 53.1 | 1 | ref. | Χ^2^ _df=2_ =1.1047  *P* = 0.5756 |
|  | AG | 150 | 39.2 | 176 | 40.6 | 0.97 | 0.73-1.29 |  |
|  | GG | 31 | 8.1 | 27 | 6.2 | 1. 30 | 0.76-2.25 |  |
| **HWE** |  | *P*_HWE_ = 0.6711 | | *P*_HWE_ = 0.3827 | |  |  |  |

SNP alleles are reported in the **Forward** orientation in agreement with the dbSNP database; **reference genotype** - the most frequent homozygote in the control group; Abbreviations: OR, odds ratio; CI, confidence intervals, HWE, test for Hardy-Weinberg equilibrium;

**Supplementary Table S6**. Genotype distribution of the *HAVCR2* (5q33.3) single nucleotide polymorphisms (SNPs) in NSCLC patients and controls.

| ***HAVCR2***  **(-strand)** | | **Patients**  **n = 383** | | **Controls**  **n = 433** | | **OR** | **95% CI** | **Patients vs. Controls** |
| --- | --- | --- | --- | --- | --- | --- | --- | --- |
|  |  | n | % | n | % |  |  |  |
| **10057302 C>A**  -1516 | CC | 353 | 92.4 | 401 | 92.8 | 1 | ref. | Χ^2^ _df=2_ = 1.9920  *P* = 0.3694 |
|  | CA | 29 | 7.6 | 29 | 6.7 | 1.14 | 0.67-1.93 |  |
|  | AA | 0 | 0 | 2 | 0.5 | 0.23 | 0.01-4.75 |  |
| **HWE** |  | *P*_HWE_ = 0.4406 | | *P*_HWE_ = 0.0728 | |  |  |  |
| **rs1036199** **C>A**  c.419 exon 3  (p.Arg140Leu) | AA | 245 | 64.3 | 277 | 64.1 | 1 | ref. | Χ^2^ _df=2_ = 1.678  *P* = 0.9195 |
|  | AC | 119 | 31.2 | 138 | 31.9 | 0.98 | 0.72-.1.32 |  |
|  | CC | 17 | 4.5 | 17 | 3.9 | 1.13 | 0.57-2.24 |  |
| **HWE** |  | *P*_HWE_ = 0.6006 | | *P*_HWE_ = 0.9710 | |  |  |  |

SNP alleles are reported in the **Forward** orientation in agreement with the dbSNP database; **reference genotype** - the most frequent homozygote in the control group; Abbreviations: OR, odds ratio; CI, confidence intervals, HWE, test for Hardy-Weinberg equilibrium;

**Supplementary Table 7.** Genotype distribution of the *PDCD1* (2q37.3) single nucleotide polymorphisms (SNPs) in LUSC and controls.

| ***PDCD1***  **(-strand)** | | **Patients**  **n = 116** | | **Controls**  **n = 433** | | **OR** | **95% CI** | **Patients vs. Controls** |
| --- | --- | --- | --- | --- | --- | --- | --- | --- |
|  |  | n | % | n | % |  |  |  |
| **rs36084323* C>T**  535 bp of *PDCD1*  **PD-1.1 (-606)** | CC | 111 | 95.7 | 415 | 95.8 | 1 | ref. | Χ^2^ _df=1_ = 0.0053  *P* = 0.9417 |
|  | CT | 5 | 4.3 | 18 | 4.2 | 1.11 | 0.42-2.94 |  |
| **HWE** |  | *P*_HWE_ = 0.8125 | | *P*_HWE_ = 0.6587 | |  |  |  |
| **rs7421861 A>G**  c.77-218 intron 1 | AA | 49 | 42.2 | 182 | 42.0 | 1 | ref. | Χ^2^ _df=2_ = 0.7446  *P* = 0.6892 |
|  | AG | 51 | 44.0 | 203 | 46.9 | 0.93 | 0.60-1.45 |  |
|  | GG | 16 | 13.8 | 48 | 11.1 | 1.25 | 0.66-2.38 |  |
| **HWE** |  | *P*_HWE_ = 0.6413 | | *P*_HWE_ = 0.4419 | |  |  |  |
| **rs11568821 C>T**  c.627+189 intron 4  **PD-1.3** | CC | 95 | 81.9 | 362 | 83.6 | 1 | ref. | Χ^2^ _df=2_ = 2.9886  *P* = 0.2244 |
|  | CT | 17 | 14.7 | 66 | 15.2 | 1.00 | 0.56-1.77 |  |
|  | TT | 4 | 3.4 | 5 | 1.2 | 3.11 | 0.88-11.02 |  |
| **HWE** |  | *P*_HWE_ = 0.0104 | | *P*_HWE_ = 0.3175 | |  |  |  |
| **rs2227981 A>G**  c.804 exon 5 (p.Ala268=) **PD-1.3** | GG | 38 | 32.8 | 147 | 33.9 | 1 | ref. | Χ^2^ _df=2_ = 0.1026  *P* = 0.9500 |
|  | GA | 59 | 50.9 | 213 | 49.2 | 1.07 | 0.68-1.69 |  |
|  | AA | 19 | 16.4 | 73 | 16.9 | 1.02 | 0.55-1.88 |  |
| **HWE** |  | *P*_HWE_ = 0.6257 | | *P*_HWE_ = 0.7798 | |  |  |  |
| **rs10204525 C>T** c.*889 (3'UTR) **PD-1.6** | CC | 93 | 80.2 | 340 | 78.5 | 1 | ref. | Χ^2^ _df=2_ = 0.5944  *P* = 0.7429 |
|  | CT | 22 | 19.0 | 85 | 19.6 | 0.96 | 0.57-1.61 |  |
|  | TT | 1 | 0.9 | 8 | 1.8 | 0. 66 | 0.11-3.70 |  |
| **HWE** |  | *P*_HWE_ = 0.8091 | | *P*_HWE_ = 0.3249 | |  |  |  |

SNP alleles are reported in the **Forward** orientation in agreement with the dbSNP database; **reference genotype** - the most frequent homozygote in the control group; Abbreviations: OR, odds ratio; CI, confidence intervals, HWE, test for Hardy-Weinberg equilibrium; *LD z rs2227982;

**Supplementary Table S8**. Genotype distribution of the *CD274* (9p24.1) single nucleotide polymorphisms (SNPs) in LUSC patients and controls.

| ***CD274***  **(+strand)** | | **Patients**  **n = 116** | | **Controls**  **n = 433** | | **OR** | **95% CI** | **Patients vs. Controls** |
| --- | --- | --- | --- | --- | --- | --- | --- | --- |
|  |  | n | % | n | % |  |  |  |
| **rs822335 T>C**  2.3 kb 5’ of  *CD274* | CC | 37 | 31.9 | 178 | 41.1 | 1 | ref. | Χ^2^ _df=2_ = 3.6096  *P* = 0.1650 |
|  | CT | 64 | 55.2 | 199 | 46.0 | 1.54 | 0.98-2.41 |  |
|  | TT | 15 | 12.9 | 56 | 12.9 | 1.31 | 0.67-2.53 |  |
| **HWE** |  | *P*_HWE_ = 0.1193 | | *P*_HWE_ = 0.9739 | |  |  |  |
| **rs10815225 G>C**  (-114) 5bp 5' of  *CD274* | GG | 92 | 79.3 | 345 | 79.7 | 1 | ref. | Χ^2^ _df=2_ = 0.4974  *P* = 0.7798 |
|  | GC | 22 | 19.0 | 76 | 16.6 | 1.10 | 0.65-1.85 |  |
|  | CC | 2 | 1.0 | 12 | 2.8 | 0.75 | 0.19-2.96 |  |
| **HWE** |  | *P*_HWE_ = 0.6123 | | *P*_HWE_ = 0.0034 | |  |  |  |
| **rs17718883 C>G**  c.437 exon 4 (p.Pro146Arg) | CC | 110 | 99.1 | 423 | 99.5 | 1 | ref. | Χ^2^ _df=1_ = 0.2923  *P* = 0.5888 |
|  | GC | 1 | 0.9 | 2 | 0.50 | 2.30 | 0.30-17.60 |  |
| **HWE** |  | *P*_HWE_ = 0.9620 | | *P*_HWE_ = 0.9612 | |  |  |  |
| **rs2297136G>A**  c.*93G>A exon 7 (3'UTR) | GG | 30 | 26.1 | 112 | 25.9 | 1 | ref. | Χ^2^ _df=2_ = 0.0382  *P* = 0.9811 |
|  | GA | 61 | 53.0 | 233 | 53.9 | 0.97 | 0.60-1.58 |  |
|  | AA | 24 | 20.9 | 87 | 20.1 | 1.03 | 0.57-1.88 |  |
| **HWE** |  | *P*_HWE_ = 0.4941 | | *P*_HWE_ = 0.0871 | |  |  |  |
| **rs4143815 G>C**  c.*395 exon 7 (3'UTR) | GG | 50 | 43.1 | 206 | 47.6 | 1 | ref. | Χ^2^ _df=2_ = 8.6590  *P* = 0.0132 |
|  | GC | 44 | 37.9 | 187 | 43.2 | 0.97 | 0.62-1.52 |  |
|  | CC | 22 | 19.0 | 40 | 9.2 | 2.27 | 1.25-4.14 |  |
| **HWE** |  | *P*_HWE_ = 0.0362 | | *P*_HWE_ = 0.7938 | |  |  |  |
| **rs4742098 A>G** c.*2635A exon 7 (3'UTR) | AA | 61 | 52.6 | 230 | 53.1 | 1 | ref. | Χ^2^ _df=2_ =6.2495  *P* = 0.0439 |
|  | AG | 40 | 34.5 | 176 | 40.6 | 0.86 | 0.55-1.34 |  |
|  | GG | 15 | 12.9 | 27 | 6.2 | 2.11 | 1.07-4.18 |  |
| **HWE** |  | *P*_HWE_ = 0.0504 | | *P*_HWE_ = 0.3827 | |  |  |  |

SNP alleles are reported in the **Forward** orientation in agreement with the dbSNP database; **reference genotype** - the most frequent homozygote in the control group; Abbreviations: OR, odds ratio; CI, confidence intervals, HWE, test for Hardy-Weinberg equilibrium;

**Supplementary Table S9**. Genotype distribution of the *HAVCR2* (5q33.3) single nucleotide polymorphisms (SNPs) in LUSC patients and controls.

| ***HAVCR2***  **(-strand)** | | **Patients**  **n = 116** | | **Controls**  **n = 433** | | **OR** | **95% CI** | **Patients vs. Controls** |
| --- | --- | --- | --- | --- | --- | --- | --- | --- |
|  |  | n | % | n | % |  |  |  |
| **10057302 C>A**  -1516 | CC | 106 | 91.4 | 401 | 92.8 | 1 | ref. | Χ^2^ _df=2_ =1.0253  *P* = 0.5989 |
|  | CA | 10 | 8.6 | 29 | 6.7 | 1.34 | 0.64-2.80 |  |
|  | AA | 0 | 0.0 | 2 | 0.5 | 0.75 | 0.04-15.82 |  |
| **HWE** |  | *P*_HWE_ = 0.6276 | | *P*_HWE_ = 0.0728 | |  |  |  |
| **rs1036199** **C>A**  c.419 exon 3  (p.Arg140Leu) | AA | 79 | 68.1 | 277 | 64.1 | 1 | ref. | Χ^2^ _df=2_ = 0.6381  *P* = 0.7268 |
|  | AC | 33 | 28.4 | 138 | 31.9 | 0.84 | 0.54-.1.33 |  |
|  | CC | 4 | 3.4 | 17 | 3.9 | 0.90 | 0.31-2.61 |  |
| **HWE** |  | *P*_HWE_ = 0.8098 | | *P*_HWE_ = 0.9710 | |  |  |  |

SNP alleles are reported in the **Forward** orientation in agreement with the dbSNP database; **reference genotype** - the most frequent homozygote in the control group; Abbreviations: OR, odds ratio; CI, confidence intervals, HWE, test for Hardy-Weinberg equilibrium;

**Supplementary Table S10**. Genotype distribution of the *PDCD1* (2q37.3) single nucleotide polymorphisms (SNPs) in LUAD patients and control subjects.

| ***PDCD1***  **(-strand)** | | **Patients**  **n= 112** | | **Controls**  **n = 433** | | **OR** | **95 % CI** | **Patients vs. Controls** |
| --- | --- | --- | --- | --- | --- | --- | --- | --- |
|  |  | N | % | N | % |  |  |  |
| **rs36084323* C>T**  535 bp of *PDCD1*  **PD-1.1 (-606)** | CC | 112 | 100 | 415 | 95.8 | 1 | ref. | Χ^2^ _df=1_ = 4.8061  *P* = 0.0284 |
|  | CT | 0 | --- | 18 | 4.2 | 0.10 | 0.01-1.67 |  |
| **HWE** |  | --- | | *P*_HWE_ = 0.6587 | |  |  |  |
| **rs7421861 A>G**  c.77-218 intron 1 | AA | 50 | 44.6 | 182 | 42.0 | 1 | ref. | Χ^2^ _df=2_ = 1.5761  *P* = 0.4547 |
|  | AG | 46 | 41.1 | 203 | 46.9 | 0.83 | 0.53-1.29 |  |
|  | GG | 16 | 14.3 | 48 | 11.1 | 1.23 | 0.65-2.33 |  |
| **HWE** |  | *P*_HWE_ = 0.3138 | | *P*_HWE_ = 0.4419 | |  |  |  |
| **rs11568821 C>T**  c.627+189 intron 4  **PD-1.3** | CC | 91 | 81.3 | 362 | 83.6 | 1 | ref. | Χ^2^ _df=2_ = 2.0383  *P* = 0.3609 |
|  | CT | 21 | 18.8 | 66 | 15.2 | 1.28 | 0.75-2.19 |  |
|  | TT | 0 | 0.0 | 5 | 1.2 | 0.36 | 0.02-6.57 |  |
| **HWE** |  | *P*_HWE_ = 0.2736 | | *P*_HWE_ = 0.3175 | |  |  |  |
| **rs2227981 A>G**  c.804 exon 5 (p.Ala268=) **PD-1.3** | GG | 40 | 35.7 | 147 | 33.9 | 1 | ref. | Χ^2^ _df=2_ = 0.8579  *P* = 0.6512 |
|  | GA | 50 | 44.6 | 213 | 49.2 | 0.86 | 0.54-1.37 |  |
|  | AA | 22 | 19.6 | 73 | 16.9 | 1.11 | 0.62-2.00 |  |
| **HWE** |  | *P*_HWE_ = 0.3770 | | *P*_HWE_ = 0.7798 | |  |  |  |
| **rs10204525 C>T** c.*889 (3'UTR) **PD-1.6** | CC | 90 | 80.3 | 340 | 78.5 | 1 | ref. | Χ^2^ _df=2_ = 0.1851  *P* = 0.9116 |
|  | CT | 20 | 17.9 | 85 | 19.6 | 0.90 | 0.53-1.54 |  |
|  | TT | 2 | 1.8 | 8 | 1.8 | 1. 11 | 0.27-4.62 |  |
| **HWE** |  | *P*_HWE_ = 0.4805 | | *P*_HWE_ = 0.3249 | |  |  |  |

SNP alleles are reported in the **Forward** orientation in agreement with the dbSNP database; **reference genotype** - the most frequent homozygote in the control group; Abbreviations: OR, odds ratio; CI, confidence intervals, HWE, test for Hardy-Weinberg equilibrium *LD z rs2227982;

**Supplementary Table S11**. Genotype distribution of the *CD274* (9p24.1) single nucleotide polymorphisms (SNPs) in LUAD patients and controls.

| ***CD274***  **(+strand)** | | **Patients**  **n = 112** | | **Controls**  **n = 433** | | **OR** | **95% CI** | **Patients vs. Controls** |
| --- | --- | --- | --- | --- | --- | --- | --- | --- |
|  |  | n | % | n | % |  |  |  |
| **rs822335 T>C**  2.3 kb 5’ of  *CD274* | CC | 55 | 49.1 | 178 | 41.1 | 1 | ref. | Χ^2^ _df=2_ = 2.3438  *P* = 0.3098 |
|  | CT | 44 | 39.3 | 199 | 46.0 | 0.72 | 0.46-1.12 |  |
|  | TT | 13 | 11.6 | 56 | 12.9 | 0.77 | 0.39-1.50 |  |
| **HWE** |  | *P*_HWE_ = 0.3643 | | *P*_HWE_ = 0.9739 | |  |  |  |
| **rs10815225 G>C**  (-114) 5bp 5' of  *CD274* | GG | 82 | 73.2 | 345 | 79.7 | 1 | ref. | Χ^2^ _df=2_ = 3.3976  *P* = 0.1829 |
|  | GC | 28 | 25.0 | 76 | 17.5 | 1.56 | 0.95-2.55 |  |
|  | CC | 2 | 1.8 | 12 | 2.8 | 0.84 | 0.21-3.33 |  |
| **HWE** |  | *P*_HWE_ = 0.8255 | | *P*_HWE_ = 0.0034 | |  |  |  |
| **rs17718883 C>G**  c.437 exon 4 (p.Pro146Arg) | CC | 109 | 100 | 423 | 99.5 | 1 | ref. | Χ^2^ _df=2_ = 0.5139  *P* = 0.4735 |
|  | GC | 0 | 0.0 | 2 | 0.50 | 0.77 | 0.04-16.23 |  |
| **HWE** |  | --- | | *P*_HWE_ = 0.9612 | |  |  |  |
| **rs2297136G>A**  c.*93G>A exon 7 (3'UTR) | GG | 28 | 25.0 | 112 | 25.9 | 1 | ref. | Χ^2^ _df=2_ = 0.4327  *P* = 0.8055 |
|  | GA | 64 | 57.1 | 233 | 53.9 | 1.09 | 0.66-1.79 |  |
|  | AA | 20 | 17.9 | 87 | 20.1 | 0.92 | 0.49-1.74 |  |
| **HWE** |  | *P*_HWE_ = 0.1155 | | *P*_HWE_ = 0.0871 | |  |  |  |
| **rs4143815 G>C**  c.*395 exon 7 (3'UTR) | GG | 48 | 42.9 | 206 | 47.6 | 1 | ref. | Χ^2^ _df=2_ =0.8415  *P* = 0.6566 |
|  | GC | 52 | 46.4 | 187 | 43.2 | 1.19 | 0.77-1.85 |  |
|  | CC | 12 | 10.7 | 40 | 9.2 | 1.31 | 0.65-2.67 |  |
| **HWE** |  | *P*_HWE_ = 0.7067 | | *P*_HWE_ =0.7938 | |  |  |  |
| **rs4742098 A>G** c.*2635A exon 7 (3'UTR) | AA | 63 | 56.3 | 230 | 53.1 | 1 | ref. | Χ^2^ _df=2_ = 0.3827  *P* = 0.8259 |
|  | AG | 42 | 37.5 | 176 | 40.6 | 0.87 | 0.57-1.35 |  |
|  | GG | 7 | 6.3 | 27 | 6.2 | 0.99 | 0.42-2.33 |  |
| **HWE** |  | *P*_HWE_ = 1.000 | | *P*_HWE_ =0.3827 | |  |  |  |

SNP alleles are reported in the **Forward** orientation in agreement with the dbSNP database; **reference genotype** - the most frequent homozygote in the control group; Abbreviations: OR, odds ratio; CI, confidence intervals, HWE, test for Hardy-Weinberg equilibrium;

**Supplementary Table S12**. Genotype distribution of the *HAVCR2* (5q33.3) single nucleotide polymorphisms (SNPs) in LUAD patients and controls.

| ***HAVCR2***  **(-strand)** | | **Patients**  **n = 112** | | **Controls**  **n = 433** | | **OR** | **95% CI** | **Patients vs. Controls** |
| --- | --- | --- | --- | --- | --- | --- | --- | --- |
|  |  | n | % | n | % |  |  |  |
| **10057302 C>A**  -1516 | CC | 103 | 92.0 | 401 | 92.8 | 1 | ref. | Χ^2^ _df=2_ = 0.7484  *P* = 0.6878 |
|  | CA | 9 | 8.0 | 29 | 6.7 | 1.25 | 0.58-2.68 |  |
|  | AA | 0 | 0.0 | 2 | 0.5 | 0.78 | 0.04-16.29 |  |
| **HWE** |  | *P*_HWE_ = 0.6578 | | *P*_HWE_ = 0.0728 | |  |  |  |
| **rs1036199** **C>A**  c.419 exon 3  (p.Arg140Leu) | AA | 64 | 57.7 | 277 | 64.1 | 1 | ref. | Χ^2^ _df=2_ = 1.5832  *P* = 0.4531 |
|  | AC | 42 | 37.8 | 138 | 31.9 | 1.32 | 0.85-.2.05 |  |
|  | CC | 5 | 4.5 | 17 | 3.9 | 1.35 | 0.50-3.66 |  |
| **HWE** |  | *P*_HWE_ 0.5640 | | *P*_HWE_ 0.9710 | |  |  |  |

SNP alleles are reported in the **Forward** orientation in agreement with the dbSNP database; **reference genotype** - the most frequent homozygote in the control group; Abbreviations: OR, odds ratio; CI, confidence intervals, HWE, test for Hardy-Weinberg equilibrium;

**Supplementary Table S13**. Distribution of the most frequent *PDCD1* haplotype.

| ***PDCD1*** | **Frequency [%]** | | | |
| --- | --- | --- | --- | --- |
| **Haplotype** | **Overall^*^** | **NSCLC** | **LUSC** | **LUAD** |
| **C-A-C-A-C** | 31.59 | 31.94 | 30.96 | 35.02 |
| C-G-C-G-C | 26.03 | 26.46 | 29.70 | 23.21 |
| C-A-C-G-C | 21.60 | 21.20 | 21.67 | 21.30 |
| C-A-C-A-T | 9.26 | 9.00 | 8.84 | 8.65 |
| C-G-T-G-C  Others^***^ | 9.17  2.35 | 9.65  1.76 | 7.30  1.52 | 9.93  1.89 |

rs36084323 (C>T); rs7421861 (A>G); rs11568821 (C>T); rs2227981 (A>G); rs10204525; Overall** (Controls + Patients)

Others^***^- combined haplotypes with a prevalence of less than 5% in the whole group

**Supplementary Table S14**. Distribution of the most frequent *CD274* haplotypes.

| ***CD274*** | **Frequency [%]** | | | |
| --- | --- | --- | --- | --- |
| **Haplotype** | **Overall^**^** | **NSCLC** | **LUSC** | **LUAD** |
| **C^*^-G-C-G-G-A** | 30.63 | 31.42 | 31.48 | 33.00 |
| T-G-C-G-G-A | 12.13 | 10.87 | 11.43 | 11.28 |
| T-G-C-A-C-G | 11.84 | 12.39 | 15.82 | 10.46 |
| C-G-C-A-G-A | 10.49 | 9.00 | 8.96 | 9.39 |
| T-G-C-A-G-A | 6.74 | 6.52 | 7.43 | 5.88 |
| C-G-C-G-C-G | 5.28 | 6.13 | 5.14 | 7.93 |
| C-C-C-A-C-G  Others^***^ | 5.22  17.67 | 5.83  17.85 | 4.03  15.71 | 5.32  16.73 |

rs822335 (**C**^*^>T); rs10815225 (**G**>C); rs17718883 (**C**>G); rs2297136 (**G**>A); rs4143815 (**G**>C); rs4742098 (**A**>G)

*change according to the frequency in the European population and our study; Overall^**^ (Controls + Patients); Others^***^- combined haplotypes with a prevalence of less than 5% in the whole group

**Supplementary Table S15**. Association of *CD274* haplotypes with age at diagnosis of NSCLC patients.

| ***CD274*** |  | | | |
| --- | --- | --- | --- | --- |
| **Haplotype** | **Parameter [year]** | **95% CI** | | ***P* value** |
| **C^*^-G-C-G-G-A** | ref.*^**^* |  |  |  |
| C-C-C-A-C-G | -0.35 | -1.86 | 1.15 | 0.645 |
| C-G-C-**A**-G-A | -1.51 | -2.74 | -0.28 | 0.016 |
| T-G-C-A-C-G | 1.21 | 0.07 | 2.34 | 0.037 |
| **T**-G-C-**A**-G-A | 1.98 | 0.40 | 3.56 | 0.014 |
| T-G-C-G-G-A | 0.34 | -0.77 | 1.46 | 0.546 |

rs822335 (**C**^*^>T); rs10815225 (**G**>C); rs17718883 (**C**>G); rs2297136 (**G**>A); rs4143815 (**G**>C); rs4742098 (**A**>G) *change according to the frequency in the European population and our study; ** the most frequent haplotype; p-multivariate linear regression adjusted for smoking; statistically significant (*p*<0.05); haplotypes with a frequency of less than 5 % were not analysed separately

**Supplementary Table S16**. Association of *CD274* haplotypes with age at diagnosis of LUSC patients.

| ***CD274*** |  | | | |
| --- | --- | --- | --- | --- |
| **Haplotype** | **Parameter [year]** | **95% CI** | | ***P* value** |
| **C^*^-G-C-G-G-A** | ref.*^**^* |  |  |  |
| C-C-C-A-C-G | 0.32 | -1.81 | 2.44 | 0.771 |
| C-G-C-**A**-G-A | -3.26 | -5.45 | -1.07 | 0.004 |
| T-G-C-A-C-A | -0.97 | -3.40 | 1.47 | 0.437 |
| **T**-G-C-**A**-**C**-**G** | -1.83 | -3.44 | -0.22 | 0.026 |
| T-G-C-A-G-A | 2.20 | -0.18 | 4.59 | 0.070 |
| T-G-C-G-G-A | 1.09 | -0.69 | 2.87 | 0.231 |

rs822335 (**C**^*^>T); rs10815225 (**G**>C); rs17718883 (**C**>G); rs2297136 (**G**>A); rs4143815 (**G**>C); rs4742098 (**A**>G)

*change according to the frequency in the European population and our study; ** the most frequent haplotype; *p*-multivariate

linear regression adjusted for smoking; statistically significant (*p*<0.05); haplotypes with a frequency of less than 5 % were not analysed separately

**Supplementary Table S17.** Association of *CD274* haplotypes with age at diagnosis of LUAD patients.

| ***CD274*** |  | | | |
| --- | --- | --- | --- | --- |
| **Haplotype** | **Parameter [year]** | **95% CI** | | ***P* value** |
| **C^*^-G-C-G-G-A** | ref.*^**^* |  |  |  |
| C-C-C-A-C-G | 0.81 | -1.87 | 3.48 | 0.556 |
| C-G-C-G-C-G | 0.78 | -2.03 | -3.59 | 0.587 |
| C-G-C-A-G-A | -0.91 | -3.28 | 1.46 | 0.452 |
| **T**-G-C-**A**-**C**-**G** | 6.13 | 3.42 | 8.84 | <0.001 |
| T-G-C-G-G-A | -1.91 | -4.16 | 0.33 | 0.096 |

rs822335 (**C**^*^>T); rs10815225 (**G**>C); rs17718883 (**C**>G); rs2297136 (**G**>A); rs4143815 (**G**>C); rs4742098 (**A**>G)

*change according to the frequency in the European population and our study; ** the most frequent haplotype; *p*-multivariate

linear regression adjusted for smoking; statistically significant (*p*<0.05); haplotypes with a frequency of less than 5 % were not analysed separately

**Supplementary Table S18**. Association of *PDCD1* haplotypes with the overall survival of NSCLC patients who underwent surgery.

| ***PDCD1*** |  | | | |
| --- | --- | --- | --- | --- |
| **Haplotype** | **HR** | **95% CI** | | ***P* value** |
| **C-A-C-A-C** | ref.*^*^* |  |  |  |
| C-A-C-A-T | 0.66 | 0.40 | 1.10 | 0.114 |
| C-A-C-G-C | 0.85 | 0.55 | 1.30 | 0.452 |
| C-G-C-G-C | 0.90 | 0.63 | 1.28 | 0.552 |
| C-**G**-**T**-**G**-C | 1.70 | 1.13 | 2.57 | 0.011 |

rs36084323 (C>T); rs7421861 (A>G); rs11568821 (C>T); rs2227981 (A>G); rs10204525;*the most frequent haplotype;

*p*-multivariate Cox proportional hazard models adjusted for smoking; statistically significant (*p*<0.05); haplotypes with a frequency of less than 5 % were not analysed separately


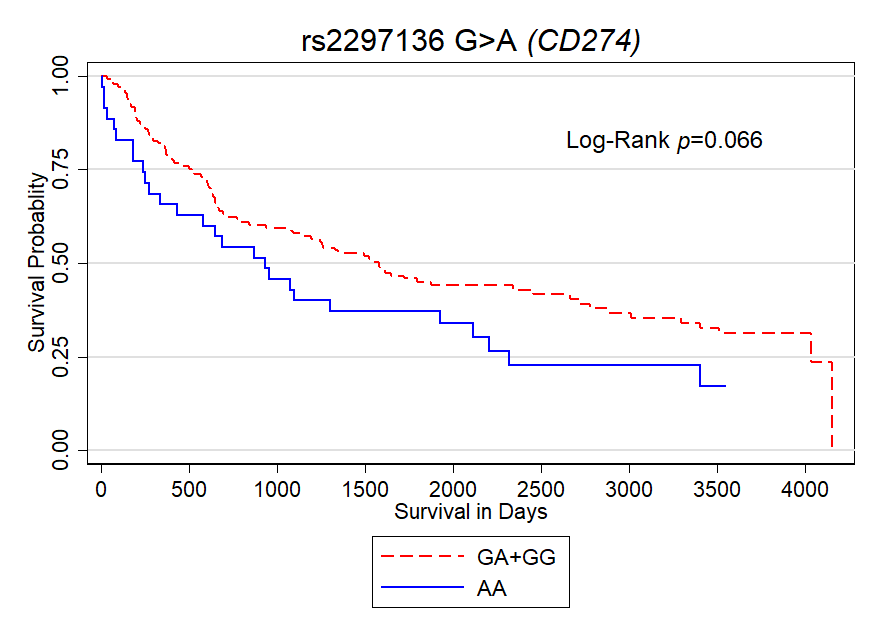


**Supplementary Figure 1**. The rs2297136 germline variant of the *CD274* gene predicts the overall survival of NSCLC patients undergoing surgery. The carriers of the G allele (GA+ GG) had a lower risk of death (HR = 0.67, 95% CI, 0.43-1.03, *P* = 0.07). After adjustment of Cox proportional hazard models for smoking and gender, the statistical significance was lost (aHR = 0.70, 95% CI, 0.45-1.09, *P* = 0.12).


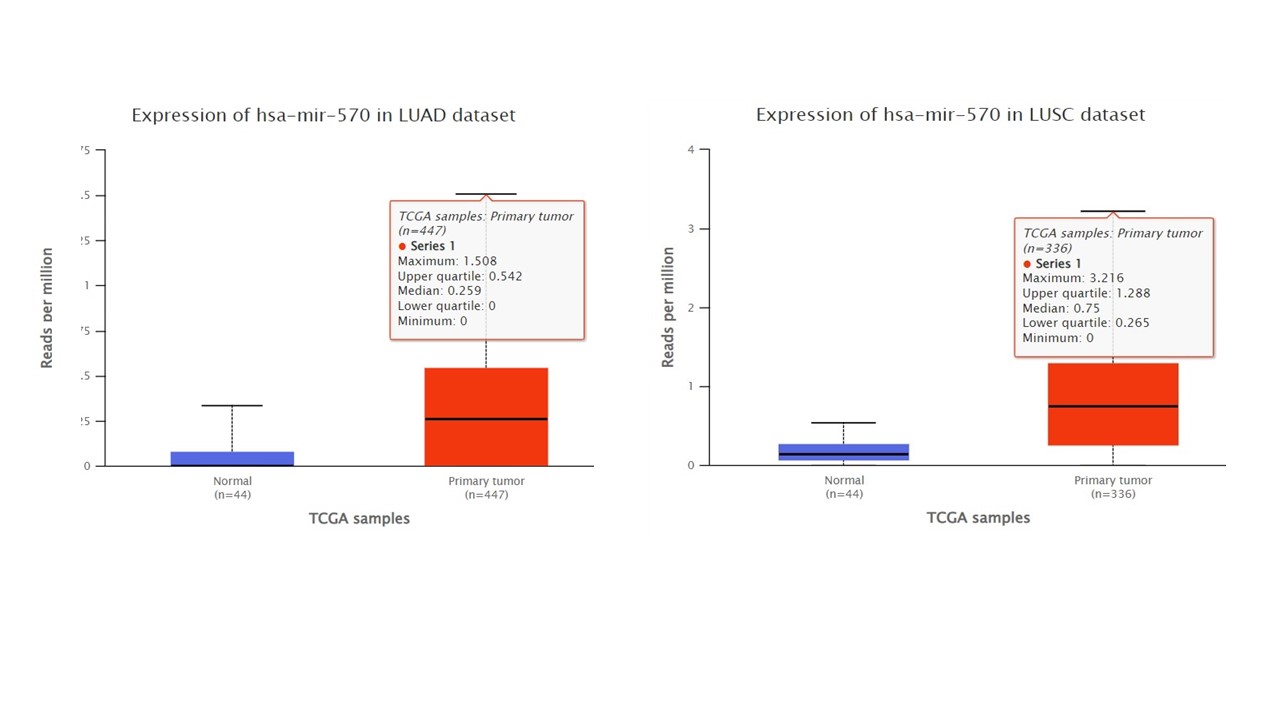


**Supplementary Figure 2**. Higher expression of the-mir 570 in LUSC in relation to LUAD.

UALCAN data analysis portal was used for the expression analysis.


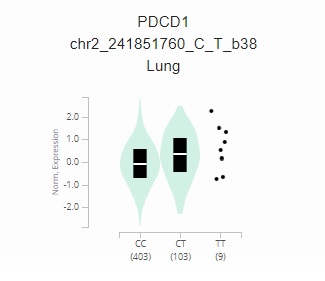


**Supplementary Figure 3.** Violin plot demonstrating a correlation between *PDCD1* gene expression and rs11568821 C>T. Allele T was associated with significantly higher expression of *PDCD1* in lung tissue (*P* = 6.6^-8^). Expression quantitative trait loci (eQTL) analysis was conducted using the Genotype-Tissue Expression (GTEx) portal (V8 release).
